# Impact of Tribological Conditions on Collagen Coating Self-Healing

**DOI:** 10.3390/ma17061341

**Published:** 2024-03-14

**Authors:** Sung-Jun Lee, Chang-Lae Kim

**Affiliations:** Department of Mechanical Engineering, Chosun University, Gwangju 61452, Republic of Korea

**Keywords:** coating, collagen, friction, self-healing, wear

## Abstract

The study examined the correlation between collagen coating damage and self-healing under various tribological conditions. It confirmed that the friction coefficient and degree of damage on the collagen coating varied based on contact and sliding conditions. The friction coefficient, measured at 0.56 for a single sliding cycle under a 350 mN normal load, demonstrated a notable decrease to 0.46 for 2295 cycles under 30 mN, further reducing to 0.15 for 90 cycles under a 20 mN normal load. As the normal load increased, the friction coefficient decreased, and with repeated sliding cycles under the same load, the coefficient also decreased. Water droplets induced a self-healing effect on collagen coating, causing wear tracks to vanish as fibers absorbed water. Severe wear tracks, with broken fibers and peeled coating, showed limited self-healing. In contrast, mild wear tracks, with compressed yet connected fibers, exhibited the self-healing phenomenon, making the wear tracks disappear. Real-time observations during 90 cycles under a 20 mN normal load highlighted the formation of mild wear tracks with intact collagen fibers, providing quantitative insights into self-healing characteristics. To preserve the self-healing effect of the collagen coating, it is essential to ensure tribological conditions during contact and sliding that prevent the disconnection of collagen fibers.

## 1. Introduction

The phenomenon in which a material damaged by an external force returns to its original shape is called self-healing. Studies on self-healing properties have been conducted using various materials such as metals, ceramics, and polymers [1,2,3,4,5]. In particular, studies on polymeric materials with self-healing properties based on chemical reactions have been conducted in various fields [6,7]. Studies have been reported to develop composites based on microcapsules containing healing materials, microvascular structures capable of supplying healing materials, or polymer materials with self-healing abilities [8,9,10,11]. Since these studies induce self-healing through chemical reactions using healing agents, it is difficult to apply them to areas related to biomaterials or areas where chemical contamination is a concern. In this respect, a method using collagen or hydrogel materials, which are biocompatible and do not require reaction with other chemicals, is required. Because collagen and hydrogel materials have swelling properties and self-healing abilities, they can be usefully applied in various fields related to medical/biomaterials [12,13,14]. In recent studies, there has been a notable advancement in the development of conductive strain sensors based on collagen and hydrogel materials, showcasing self-healing characteristics. These sensors have found application in detecting human movements and signals, demonstrating their utility in various academic domains, including self-healing sensors, electronic skins, and wearable electronics [15,16,17,18]. Especially, they are applied as a surface coating material for micro/nano systems, and a self-healing effect that restores damaged areas can be expected. In this regard, studies on the self-healing properties of collagen coatings and hydrogel coatings formed as thin films have been reported [19,20]. The friction and wear characteristics of the collagen coating and the hydrogel coating were analyzed, and the self-healing property, by which wear scars formed on the surface of the coating disappear spontaneously on contact with water, was confirmed. The self-healing mechanism was explained as the wear marks formed on the surface of the collagen and hydrogel coatings disappear as the internal structure of the coating changes due to the swelling phenomenon, in which the collagen fibers and hydrogel fibers expand due to contact with water [21]. The repeatability of the self-healing ability of collagen and hydrogel coatings was also confirmed. However, it was confirmed that collagen and hydrogel materials have relatively weak mechanical strength at the level of polymer materials, and when the contact pressure is very high, the collagen and hydrogel coatings are permanently damaged and their self-healing ability disappears. When these materials are manufactured in bulk, they exhibit healing properties that enable them to reattach even after being completely cut. However, when applied as a thin film coating to protect the surface, it is judged that the presence or absence of the self-healing effect is determined according to the degree of damage. Bulk forms of collagen and hydrogel materials, even if subjected to permanent damage in certain areas, exhibit a high potential for self-healing by reacting in other regions. However, when formed as extremely thin film coatings, collagen and hydrogel coatings fail to manifest self-healing properties in the presence of localized permanent damage. Therefore, to enable the application of thin film collagen and hydrogel coatings in diverse fields, it becomes imperative to establish the conditions under which the self-healing effect can occur. It is confirmed that there is no study result that has quantitatively evaluated whether the self-healing effect appears according to the degree of damage of the collagen coating and the hydrogel coating. That is, the minimum standards for the degree of damage or contact sliding conditions for maintaining the self-healing ability of the collage coating and the hydrogel coating have not yet been established.

In this study, we tried to confirm the minimum degree of surface damage to maintain the self-healing ability of the collagen coating. The friction and wear characteristics of the collagen coating were analyzed while adjusting the contact pressure applied to the coating surface and the number of sliding repetitions, and it was confirmed whether the damaged area became self-healing by contact with water. Through this, the minimum level of surface damage required for the self-healing effect to appear was confirmed, and the contact sliding tribological conditions were identified.

## 2. Materials and Methods

### 2.1. Specimen Preparation

**Figure 1:**
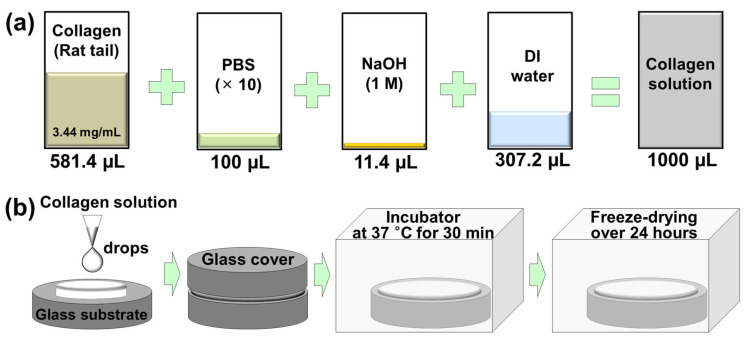
Fabrication process of (**a**) collagen solution and (**b**) collagen coating.

A collagen coating was prepared using a protein solution containing a material extracted from the tail of a rat. Figure 1 shows the process of preparing a collagen solution and forming it into a thin film coating [22]. First, a neutralized collagen solution with a concentration of 2 mg/mL was prepared by mixing 581.4 µL of protein solution with a concentration of 3.44 mg/mL, 100 µL of phosphate buffered saline (PBS) solution, 11.4 µL of 1 M sodium hydroxide (NaOH) solution, and 307.2 µL of deionized (DI) water. A small amount of collagen solution dropped on the cleaned glass substrate was placed in an incubator set at 37 °C for 30 min while covered with a glass cover to form a gel-like collagen coating. This gel-type collagen coating was freeze-dried for 24 h to completely evaporate the moisture inside the coating.

### 2.2. Experiments

To analyze the surface morphology of the collagen coating prepared in this study, a 3D laser scanning confocal microscope (3D-LSCM, VK-X200, KEYENCE. Co., Ltd., Osaka, Japan) and a scanning electron microscope (FE-SEM, 6610, JEOL, Akishima, Japan) were used. The overall three-dimensional surface morphology and surface roughness were confirmed, and the structure of collagen fibers inside the coating was analyzed.

**Figure 2:**
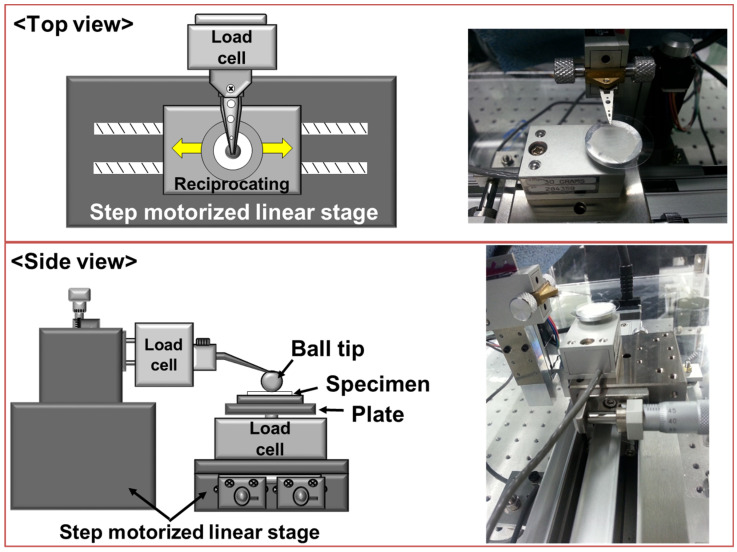
Schematic design and real photo image (top view and side view) of reciprocating-type tribotester.

To analyze the friction and wear characteristics of the collagen coating, a custom-built tribotester with reciprocating sliding motion was used as shown in Figure 2. Using a zirconia ball with a diameter of 1 mm as a counter tip, the change in the friction coefficient and the degree of damage of the collagen coating according to the contact and sliding conditions were investigated. By controlling the normal load, the level of contact pressure between the counter tip and the collagen coating could be understood, and by changing the sliding distance and speed, the variation in friction and wear characteristics of the collagen coating was analyzed. Severe contact and sliding conditions are expected to cause more severe damage to the collagen coating, and the contact and sliding conditions under which severe damage to the collagen coating do not occur were sought. Experiments were conducted by changing the experimental conditions based on the degree of damage of the collagen coating and the manifestation of the self-healing effect, using the vertical load, sliding stroke, sliding speed, and sliding cycles as variables. The experimental variables employed in this study have been summarized and presented in Table 1. All experiments were repeated three times or more for each experimental condition to secure the reliability of the results.

## 3. Results and Discussion

**Figure 3:**
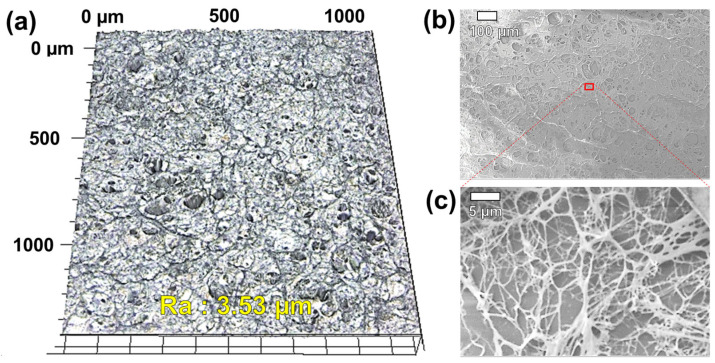
Surface images of collagen coating. (**a**) 3D-LSCM, and (**b**) low- and (**c**) high-magnification SEM images.

Figure 3 shows the surface image of the collagen coating. Figure 3a shows the 3D surface morphology of the collagen coating captured by 3D-LSCM, and Figure 3b,c show low- and high-magnification SEM images of the collagen coating, respectively. The collagen coating has a structure in which microscale collagen fibers formed using a protein solution extracted from rat tails are intricately entangled in a net shape [23]. From the three-dimensional surface morphology image taken through 3D-LSCM, it was confirmed that the average surface roughness of the collagen coating was approximately 3.53 μm. Through the SEM image enlarged with high magnification, it was confirmed that the inside of the collagen coating was randomly entangled with micro-scale thin collagen fibers in a network structure. The thickness of the collagen coating confirmed through SEM analysis of the cut side of the collagen-coated specimen was confirmed to be approximately 30 μm.

**Figure 4:**
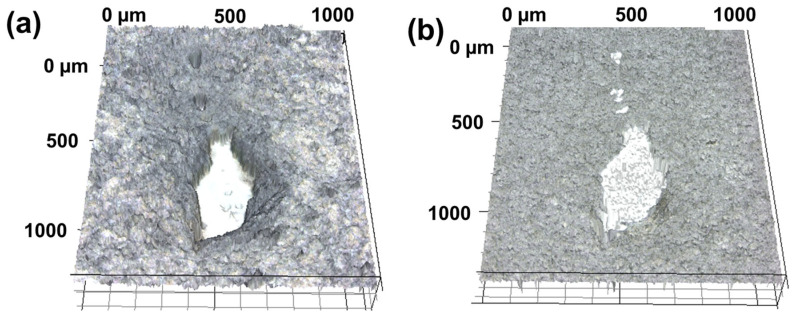
Damaged part on collagen coating (**a**) before and (**b**) after contacting with water.

Previous studies have reported that when the wear track part formed on the collagen coating comes into contact with water, a self-healing phenomenon occurs in which the wear track disappears as the collagen fibers absorb water and swell [24]. The repeatability of the self-healing effect of the collagen coating was verified, but it was necessary to verify whether the self-healing effect of the collagen fibers could be expressed even when the collagen fibers were completely cut off. The self-healing phenomenon of the collagen coating means that the wear track disappears because the collagen fibers, which have been compressed by repeated contact sliding movements, absorb water molecules and swell. If the collagen fibers are disconnected, the swelling effect of the collagen fibers is limited, and it is thought that the disconnected fiber space will remain empty. To confirm this, when the collagen-coated surface was picked and scratched with sharp tweezers, the collagen coating was dented and torn off, as shown in Figure 4a. Because the collagen coating was scratched by applying artificially strong surface contact pressure, the entangled collagen fibers were completely broken. Water droplets were dropped on the damaged area to swell the collagen fibers, and the results of observing the surface after completely drying the water are shown in Figure 4b. Although the morphology of the surface was changed due to the swelling of the collagen fibers, it was confirmed that the part that was forcibly torn off by the tweezers remained damaged without recovery. The hypothesis that the self-healing ability of the collagen coating disappears when the collagen fibers are broken was verified. In order to maintain the self-healing effect of the collagen coating, the need to understand the contact and sliding conditions in which the collagen fibers are maintained without permanent damage has emerged. In order to quantify the contact and sliding conditions, it is necessary to use a spherical ball as a counter tip and use a tribotester that can constantly adjust the normal load and sliding speed [25,26]. The friction and wear characteristics of the collagen coating according to normal load and sliding cycle were analyzed.

**Figure 5:**
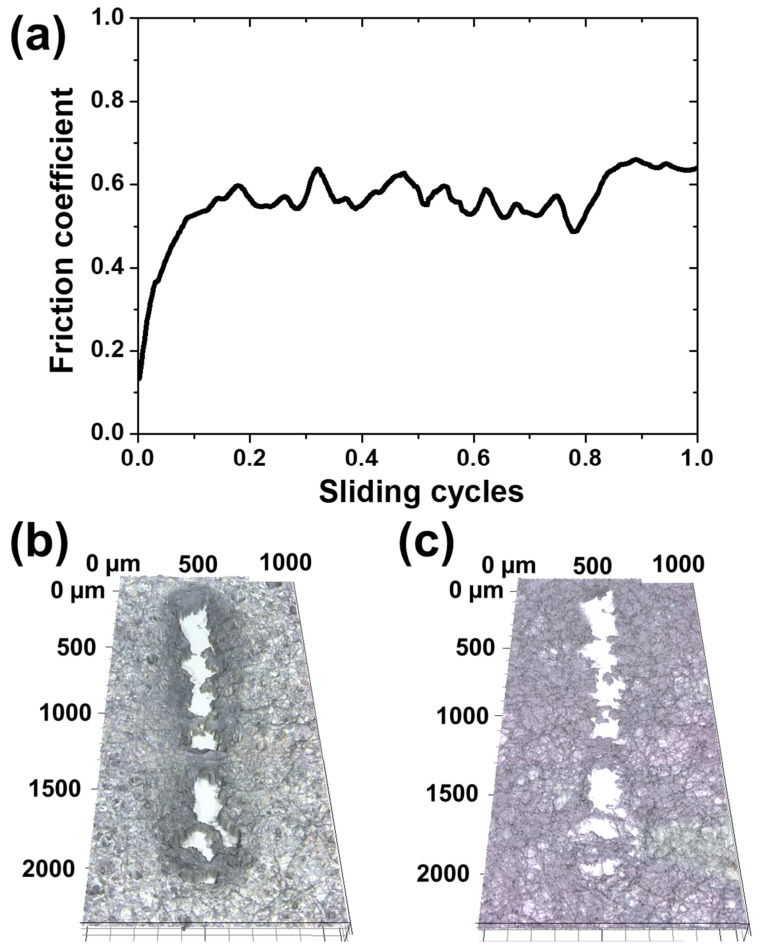
Friction and wear characteristics of collagen coatings for one cycle of sliding motion with a sliding stroke of 2 mm and a sliding speed of 1 mm/s under a vertical load of 350 mN. (**a**) Friction coefficient history and confocal microscope images of wear track (**b**) before and (**c**) after contacting with water.

After applying a vertical load of 350 mN to the collagen coating, a sliding motion of reciprocating once with a sliding stroke of 2 mm at a sliding speed of 1 mm/s was performed. Figure 5a shows the change in friction coefficient during one cycle of sliding motion. At the beginning of sliding, the friction coefficient gradually increases, then saturates around 0.55 and shows a large fluctuation. In the latter part of the slide, the friction coefficient rose slightly and exceeded 0.6, and the overall average friction coefficient was 0.56. The fluctuation of the friction coefficient throughout the sliding motion and the increase in the friction coefficient in the second half of the sliding movement are assumed to have been caused by the wear of the collagen coating. Figure 5b shows the wear track formed on the collagen-coated surface by 1 cycle of sliding motion. It can be seen that the collagen coating was completely peeled off to the extent that the substrate was exposed. In the middle of the wear track, partial collagen fibers were connected without being completely broken. Water droplets were dropped on the wear track formed in this way to check whether self-healing effect appeared. As shown in Figure 5c, the wear track that was completely torn off and damaged did not recover. It can be seen that the parts where the collagen fibers were connected in the middle of the wear track maintained the connection as it was. A load of 350 mN induces a high contact pressure, and it is considered to be a severe contact and sliding condition to the extent that the collagen fibers are completely broken and separated even in a single sliding cycle [27].

**Figure 6:**
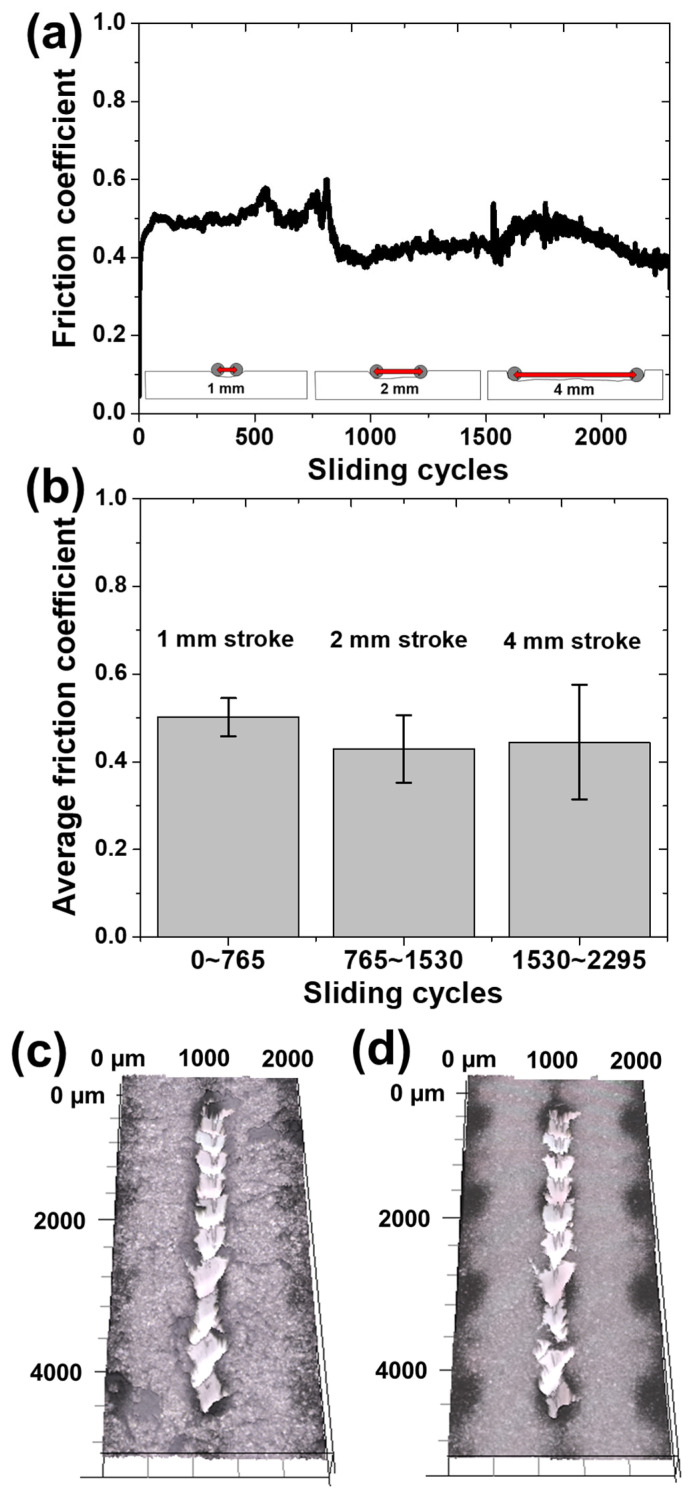
Variation in friction and wear characteristics of collagen coating with respect to the sliding stroke length. (**a**) Friction coefficient history and (**b**) average friction coefficient of collagen coating as a function of the number of sliding cycles for stroke lengths of 1 mm, 2 mm, and 4 mm. Confocal microscope images of wear track (**c**) before and (**d**) after contacting with water.

In order to greatly alleviate the contact pressure condition for the collagen coating, a sliding motion was applied under a normal load of 30 mN, which was lowered by more than 10 times compared to the severe contact condition. Instead of greatly relaxing the contact pressure condition, the sliding cycle was increased, and the sliding stroke was increased from 1 mm to 2 mm and 4 mm in the middle of the sliding motion. The sliding motion was performed for 765 cycles for each sliding stroke section, and the sliding speed was set to 4 mm/s. Figure 6a shows the change in the friction coefficient according to the total sliding cycle. First, in the case of sliding motion in the sliding stroke section of 1 mm, the friction coefficient increased from 0.4 to 0.5 at the beginning of the sliding cycle and was maintained, and then abrupt fluctuations occurred in the latter half of the sliding cycle. As the contact area between the surface area of the wear track formed on the collagen coating and the counter tip increased, the friction coefficient was thought to be increased by the repulsive force blocking the advancing direction of the tip. In addition, it is expected that serious damage to the collagen coating occurred in the section where the fluctuation of the friction coefficient occurred. After performing the sliding motion for 765 cycles with a sliding stroke of 1 mm, the sliding motion was performed with a sliding stroke of 2 mm immediately afterwards. This continuously damaged the part of the wear track corresponding to the 1 mm stroke that had been damaged to some extent, while forming a new wear track in the extended sliding stroke part. It is for the same reason that the sliding stroke was changed to 4 mm after 765 cycles of sliding motion. As the sliding stroke was changed, there was a difference in the friction coefficient for each section of the sliding stroke in the simultaneous sliding motion. In the 2 mm sliding stroke section, the friction coefficient was lower than that of the 1 mm sliding stroke, showing a level of 0.4, and as the sliding cycle increased, the friction coefficient gradually increased. The initial friction coefficient started at 0.4 in a sliding stroke of 1 mm, and as the wear track was formed, the friction coefficient increased due to the increase in the contact area. As the sliding stroke increased to 2 mm, the counter tip passed the new surface of the collagen coating, showing an initial friction coefficient of 0.4, and it is considered that the overall friction coefficient decreased. It is speculated that the reason why the friction coefficient did not increase rapidly in the newly increased 1 mm section of the 2 mm wear track is that the wear particles separated from the collagen coating by 765 cycles of sliding motion in the 1 mm sliding stroke adhered to the surface of the counter tip, causing a friction reduction effect. However, in the 1 mm sliding stroke section where the wear track was first formed in the 2 mm sliding stroke, the collagen coating was more damaged as the cumulative sliding motion exceeded 1000 cycles. It seems that the friction coefficient increased due to the increase in the contact area as the large wear track was formed in the newly contacted sliding stroke section of the remaining 1 mm. After sliding for 1530 cycles, when the sliding stroke was increased to 4 mm, the friction coefficient rapidly increased to a level of 0.5 or more, and then decreased after about 1700 cycles. In the already damaged 1 mm sliding stroke, collagen fibers were completely ripped off, which is expected to have accelerated the damage of the collagen coating. Due to the modified sliding stroke, there was a difference in surface height inside the wear track. It is believed that the newly formed wear tracks on the 2 mm and 4 mm sliding strokes were also torn off due to the shearing action caused by the frictional force acting horizontally on the surface. In this process, it seems that the friction coefficient rapidly rose and then rapidly decreased as it came into contact with the glass surface, which was the substrate. As shown in Figure 6b, when comparing the average friction coefficient for each sliding stroke section, the friction coefficients for sliding strokes of 1 mm, 2 mm, and 4 mm were 0.50, 0.43, and 0.44, respectively. The highest value was reached at 1 mm sliding stroke and the lowest value at 2 mm sliding stroke. The average friction coefficient over the entire experiment was measured to be approximately 0.46. As shown in Figure 6c, after a total of 2295 cycles of sliding, the wear tracks formed on the collagen-coated surface were completely torn off, and the substrate surface was also damaged. This was observed after dropping water droplets on completely damaged traces of the collagen-coated surface. As shown in Figure 6d, it can be seen that the collagen fibers were completely broken, and the collagen coating was torn off, leaving severe wear tracks. Even if the normal load was greatly reduced and the contact pressure condition was alleviated, it can be judged that the collagen fibers were permanently damaged if the number of sliding cycles was high [28].

**Figure 7:**
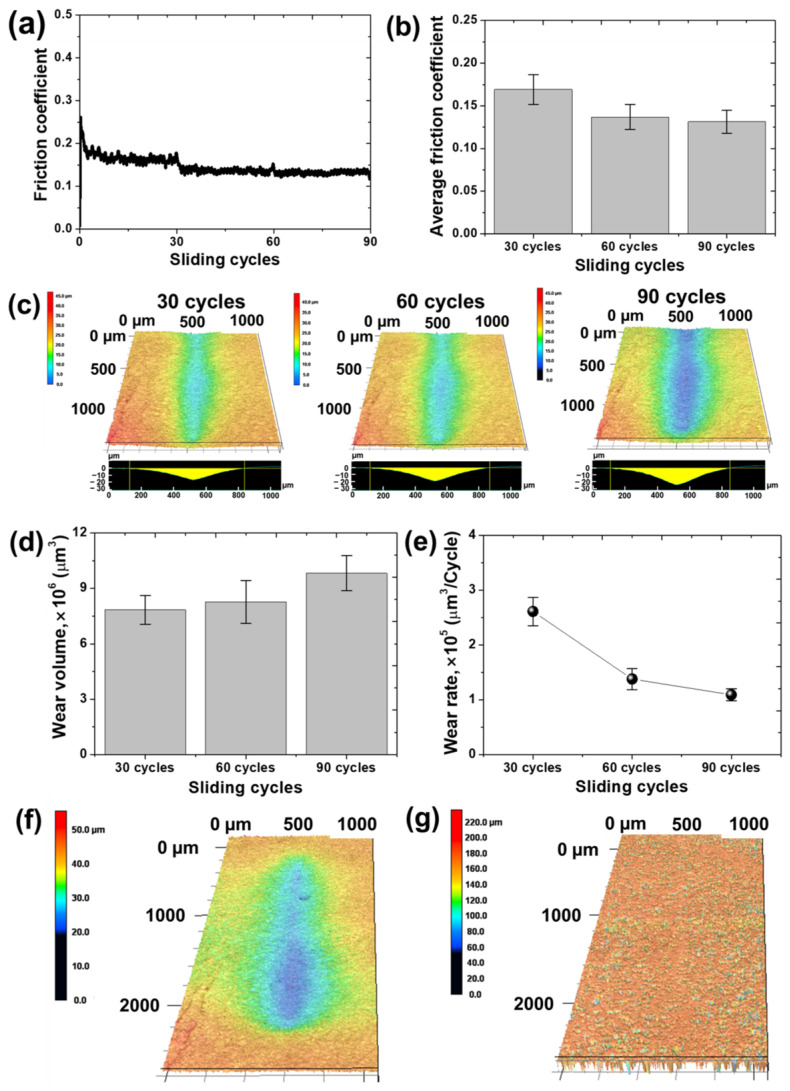
Variation in friction and wear characteristics of collagen coating with respect to the number of sliding movements. (**a**) Friction coefficient history, (**b**) average friction coefficient, (**c**) confocal microscope images of wear track, (**d**) wear volume, (**e**) wear rate. Confocal microscope images of wear track (**f**) before and (**g**) after self-healing.

After applying a normal load of 20 mN to the collagen coating to further alleviate the contact pressure condition, a sliding friction motion was performed. In addition, in order to observe the formation process of the wear track in real time and perform the experiment only until the collagen fibers were completely broken, a friction test was performed with a custom-built tribotester mounted on the 3D-LSCM. The wear track formed on the surface of the collagen coating was observed every 30 cycles, and the change in friction coefficient was analyzed in real time, as shown in Figure 7a. When a normal load of 20 mN was applied, the initial sliding motion started with a friction coefficient of approximately 0.26. After dropping rapidly below 0.2, the friction coefficient was maintained at approximately 0.18 and then gradually decreased. After observing the wear track that formed on the collagen coating at the point of 30 cycles, an additional sliding friction motion was performed again for 30 cycles, and as a result, the friction coefficient was maintained at 0.14, and this value was maintained for a total of 90 cycles thereafter. Figure 7b–e show the average friction coefficient, 2D profile and 3D surface images of the wear track, wear volume and wear rate measured every 30 cycles of sliding motion, respectively. The average friction coefficient values measured for each 30, 60, and 90 cycles of sliding motion were 0.17, 0.14, and 0.13, respectively, and tended to decrease as the sliding cycle increased. The average friction coefficient in the entire experiment was approximately 0.15. In the case of the wear track measured every 30 cycles, the width and depth of the wear track increased as the sliding cycle increased, as shown in Figure 7c. As a result of calculating and measuring the wear volume through 2D profile image analysis of the wear track, we found that it was 7.85 × 10^6^ μm^3^, 8.28 × 10^6^ μm^3^, and 9.82 × 10^6^ μm^3^ for 30, 60, and 90 cycles of sliding motion, respectively. As the sliding motion was repeated, it was a natural result that the amount of accumulated wear increased. However, when compared with the wear volume per cycle, that is, the wear rate, as the sliding cycle increased by 30 cycles, it was confirmed that the wear rate gradually decreased to 2.62 × 10^5^ µm^3^/cycle, 1.38 × 10^5^ µm^3^/cycle, and 1.09 × 10^5^ µm^3^/cycle, respectively. This means that the rate of increase in the amount of wear was low compared to the increase in the number of sliding cycles. In the initial contact sliding motion, the collagen coating was compressed to form a wear track, but the collagen coating was not completely torn off within at least 90 cycles. This is to keep the amount of wear relatively small. However, when contact and sliding conditions became severe due to a further increase in the sliding cycle or an increase in the normal load, the collagen coating was completely peeled off and the amount of wear increased rapidly. After sliding for 90 cycles, it was found that the collagen fibers were much compressed from the measured width and depth of the wear track, and it was judged that further sliding would cause permanent damage to the collagen coating, so the experiment was stopped. Figure 7f shows a 3D image of the wear tracks with the wear tracks not completely peeled off. It showed the shape of a deep and wide wear track, and it can be seen that the wear track was deeply compressed over 20 μm. When water drops were dropped on the finally formed wear track, the deep and wide wear track completely disappeared and self-healed to a flat surface, as shown in Figure 7g. When the collagen fibers were not completely cut off, but only compressed, the collagen fibers absorbed water molecules and swelled to restore the compressed wear track area. The hypothesis was verified that when the contact pressure and sliding conditions were severe, the collagen fibers were broken and the self-healing effect disappeared as the coating was peeled off, and the self-healing effect appeared only when the collagen fibers were connected to each other.

**Figure 8:**
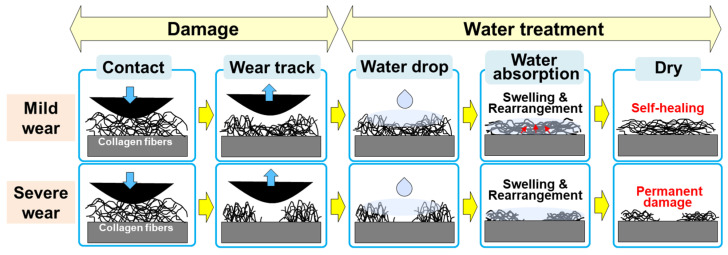
Schematic diagram of the mechanism of the self-healing effect depending on the degree of damage formed in the collagen coating.

Figure 8 shows a schematic diagram explaining the mechanism of the self-healing effect according to the degree of damage of the collagen coating. In this study, when a normal load of 20 mN was applied and sliding motion was performed for 90 cycles, the collagen fibers in the collagen coating did not break and a mild wear track was formed in a compressed state, and the collagen fibers absorbed water molecules and swelled. As the collagen fibers were rearranged, a self-healing phenomenon occurred in which the wear track disappeared [29]. On the other hand, under severe contact and sliding conditions, such as when a normal load of 350 mN was applied and 2295 cycles of sliding motion were performed under a normal load of 30 mN, the collagen fibers were broken, the coating peeled off, and a severe wear track was formed. However, even in contact with water droplets, the swelling and realignment of collagen fibers were limited, so the wear tracks did not self-heal and permanent damage remained. Through this study, it was confirmed that in order to maintain the self-healing effect of the collagen coating, permanent damage resulting from breaking the collagen fibers should not occur. In addition, the normal load and sliding cycle conditions required for maintaining the connection without the breakage of the collagen fibers were identified. 

## 4. Conclusions

In this study, a thin-film collagen coating was formed using a protein solution extracted from the tail of a rat. The inside of the collagen coating had a structure in which thin collagen fibers were intricately entangled in the form of a net. The friction and wear characteristics of collagen coatings were evaluated under various normal-load and sliding conditions. The self-healing phenomenon of the wear track formed on the surface of the collagen coating was observed according to each contact and sliding motion condition, and the conditions for the self-healing effect of the collagen coating were confirmed. The average friction coefficient was measured as 0.56 for 1 cycle of sliding under a normal load of 350 mN, 0.46 for 2295 cycles of sliding under 30 mN, and 0.15 for 90 cycles of sliding under 20 mN. As the normal load decreased, the friction coefficient generally decreased. Under the same normal load, the friction coefficient and wear rate tended to decrease as the number of sliding cycles increased. In the experiment in which the sliding motion was performed while changing the length of the sliding stroke, the transition period of the friction coefficient was clearly shown for each sliding stroke section. In the case of the sliding motion being performed for 90 cycles under a normal load of 20 mN while observing the degree of wear track formation every 30 cycles in real time, the collagen fibers were not damaged and a mild wear track was formed, whereas in all other experiments, a severe wear track was formed in which the collagen fibers were completely broken and the coating peeled off. In the case of severe wear tracks, the self-healing effect of the collagen coating did not emerge, whereas the mild wear tracks self-healed in contact with water. Therefore, through this study, the conditions of contact and sliding motion required to maintain the self-healing properties of the collagen coating were identified without completely damaging the collagen fibers. This study anticipates progress in the realm of bio-inspired coatings, capitalizing on the specifically tailored self-healing attributes of collagen coatings to enhance durability across varied applications. The potential impacts span crucial areas like medical devices, wearable technologies, and micro/nano systems, ensuring prolonged functionality with minimal maintenance. A deep understanding of collagen coatings’ self-healing mechanism provides valuable insights, fostering innovative solutions in resilient biomaterial development and contributing to advancements in biomedical engineering.

## Figures and Tables

**Table 1:** Experimental conditions.

Tribotest (Reciprocating Type)	
Tip material (diameter)	Zirconia ball (1 mm)
Normal load	20 mN, 30 mN, 350 mN
Sliding speed	1 mm/s, 4 mm/s
Sliding stroke	1, 2, 4 mm
Sliding cycle	1 cycle, 90 cycles, 2295 cycles

## Data Availability

The data presented in this study are available upon reasonable request from the corresponding author.
